# Small molecule FTO inhibitor MO-I-500 protects differentiated SH-SY5Y neuronal cells from oxidative stress

**DOI:** 10.3389/fnmol.2025.1736173

**Published:** 2026-01-12

**Authors:** Denise Greco, Zuzana Čočková, Debanjan Das, Akash S. Mali, Jiří Novotný, Mark J. Olsen, Petr Telenský

**Affiliations:** 1 Department of Physiology, Faculty of Science, Charles University, Prague, Czechia; 2 Imaging Methods Core Facility at BIOCEV, Faculty of Science, Charles University, Vestec, Czechia; 3 Department of Pharmacology, First Faculty of Medicine, Charles University and General University Hospital, Prague, Czechia; 4 Department of Pharmaceutical Sciences, Midwestern University, Campus Glendale, Glendale, AZ, United States; 5 Pharmacometrics Center of Excellence, Midwestern University, Downer’s Grove, IL, United States; 6 International Clinical Research Center of St. Anne’s University Hospital Brno, Dementia Research Group, Brno, Czechia

**Keywords:** aging, FTO inhibition, m6A, neuroprotection, oxidative stress, ROS

## Abstract

**Introduction:**

Oxidative stress is a central driver of brain aging, impairing cellular function and increasing susceptibility to neurodegenerative diseases. Recent studies suggest that the RNA demethylase FTO regulates N6-methyladenosine (m6A) RNA modification, a key pathway in modulating oxidative stress in the brain. However, the precise mechanisms underlying FTO’s role remain unclear. This study examines the neuroprotective potential of MO-I-500, a small-molecule FTO inhibitor, against oxidative stress induced by tert-butyl hydroperoxide (TBHP) in neuron-like SH-SY5Y cells differentiated with retinoic acid and BDNF (dSH-SY5Y).

**Methods:**

dSH-SY5Y cells were treated with MO-I-500 alone for 72 h or with TBHP alone for 24 h. Alternatively, cells were pretreated with 1 μM MO-I-500 for 48 h, followed by co-treatment with MO-I-500 and 25 or 50 μM TBHP for an additional 24 h, for a total treatment duration of 72 h. Cellular metabolism was assessed using a Seahorse XF MitoStress assay, and oxidative stress markers, including ROS and superoxide levels, were quantified with DCFDA and MitoSOX probes. ATP content was measured using a bioluminescence assay.

**Results:**

FTO inhibition by MO-I-500 induced a metabolic shift toward an energy-efficient state, enhancing cellular resilience to oxidative stress. Pretreatment significantly reduced TBHP-induced oxidative damage, lowering intracellular ROS levels and preserving ATP content.

**Conclusion:**

Together with our previous findings demonstrating the protective effects of MO-I-500 in astrocytes and recent studies supporting the importance of astrocyte function in neurodegeneration, these results suggest a dual protective role of MO-I-500 in neurons and astrocytes. This dual action positions MO-I-500 as a promising therapeutic strategy to mitigate oxidative damage and reduce the risk of neurodegenerative diseases, including Alzheimer’s disease.

## Graphical abstract

**Figure:**
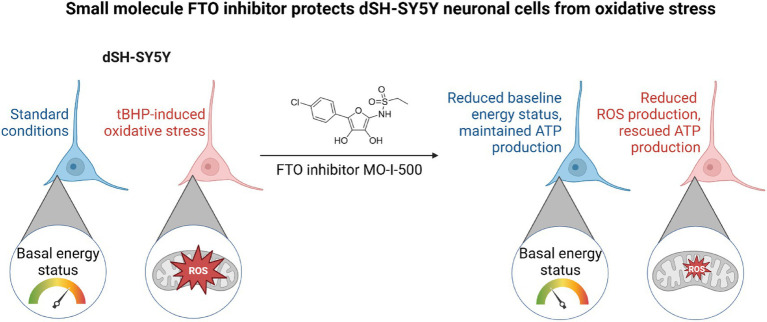

## 1 Introduction

Oxidative stress plays a central role in aging and age-related diseases, contributing to cellular damage through the accumulation of reactive oxygen species (ROS) (Giorgi et al., 2018). In brain cells, oxidative imbalance is particularly detrimental due to the high metabolic activity and energy demands of neurons, which rely heavily on mitochondrial function (Chen et al., 2023). Mitochondria are both a major source and target of ROS, producing superoxide as a byproduct of the electron transport chain (ETC) activity (Kandola et al., 2015). Under normal conditions, antioxidant systems maintain redox homeostasis, but excessive ROS can overwhelm these defenses, damaging mitochondrial DNA, proteins, and lipids (Garza-Lombó et al., 2020). This leads to disrupted mitochondrial dynamics, decreased membrane potential, and impaired ATP production (Yang et al., 2024). Chronic oxidative stress exacerbates mitochondrial dysfunction, creating a cycle that accelerates neuronal aging and degeneration (De Gaetano et al., 2021).

Aβ accumulation further amplifies oxidative damage through a feedback loop that promotes both ROS production and plaque formation, contributing to neurodegenerative progression (Chen et al., 2012). Targeting early oxidative stress and mitochondrial dysfunction thus represents a promising therapeutic strategy in neurodegeneration. In parallel, recent research highlights the importance of RNA epitranscriptomic regulation, particularly N6-methyladenosine (m6A), in neuronal health and aging (Chokkalla et al., 2020; Wu et al., 2020; Shafik et al., 2021; Minhas et al., 2024). The m6A demethylase FTO has been implicated in Alzheimer’s disease pathogenesis and oxidative stress responses, including modulation of Tau signaling and redox balance (Keller et al., 2011; Li et al., 2018; Filipovská et al., 2024; Liu et al., 2024; Song et al., 2024).

We previously demonstrated that MO-I-500, a small-molecule FTO inhibitor, protects astrocytic cells from oxidative and metabolic damage (Cockova et al., 2021). Although the efficacy in reducing demethylase activity has not been directly tested in this study, MO-I-500 is a well-characterized FTO inhibitor (Zheng et al., 2014; Singh et al., 2016; Cockova et al., 2021). Importantly, MO-I-500 crosses the blood–brain barrier via an SVCT2-mediated mechanism, making it a promising candidate for modulating FTO activity in the CNS (Zheng et al., 2014). However, its protective effects in neuronal cells remain uncharacterized.

In this study, we investigated the effects of MO-I-500 in retinoic acid (RA) and BDNF-differentiated SH-SY5Y neuronal cells (dSH-SY5Y) under normal and oxidative stress conditions. We show that FTO inhibition induces a metabolic shift toward an energy-efficient metabolic state under basal conditions, while promoting mitochondrial resilience and reducing ROS under oxidative stress, suggesting a neuroprotective mechanism.

## 2 Materials and methods

### 2.1 Cell cultures and differentiation

The human neuroblastoma SH-SY5Y cell line was obtained from the European Collection of Authenticated Cell Cultures (ECACC). Cells were maintained in Dulbecco’s Modified Eagle’s Medium-high glucose (DMEM; D6429-500ML, Sigma-Aldrich) mixed 1:1 (v/v) with Nutrient Mixture F-12 Ham (N4888-500ML, Sigma-Aldrich). The medium was supplemented with 10% fetal bovine serum (FBS; Gibco™ 10,270,106) and 1% antibiotic solution (A5955, Sigma-Aldrich). Cells were incubated at 37 °C in a humidified atmosphere with 5% CO2 and sub-cultured twice weekly upon reaching 70–80% confluence.

Neural differentiation was induced using a two-step protocol. Cells were first treated with 10 μM all-trans retinoic acid (RA) in low-serum medium (1% FBS) for 48 h. The media was then replaced with fresh RA-containing medium, and cells were incubated for an additional 72 h. Following the RA treatment, cells were switched to serum-free medium supplemented with 50 ng/mL brain-derived neurotrophic factor (BDNF; B3795, Sigma-Aldrich) and incubated for 72 h. Optimization of the differentiation protocol is shown in Supplementary Figure S2.

### 2.2 Cell viability assay

The MTT [3-(4,5-dimethylthiazol-2-yl)-2,5-diphenyl tetrazolium bromide] assay was used to assess cell viability. This colorimetric assay measures the reduction of MTT to an insoluble formazan product by mitochondria in viable cells.

To determine the optimal concentration of MO-I-500 for SH-SY5Y treatment, undifferentiated cells were seeded in 96-well plates at a density of 3 × 10⁴ cells per well and differentiated using RA and BDNF. Cells were initially exposed to 10 μM RA in medium containing 1% FBS for 48 h. After medium replacement with fresh RA-containing medium, the cells were maintained in culture for an additional 72 h. Subsequently, cells were transferred to serum-free medium supplemented with 50 ng/mL BDNF and maintained for 72 h. Differentiated SH-SY5Y (dSH-SY5Y) cells were then treated with MO-I-500 at concentrations of 0.3, 0.7, 1, 3, 5, 7, 10, and 25 μM for 72 h.

Similarly, to determine the concentration of tert-butyl hydroperoxide (TBHP) needed to induce oxidative stress, dSH-SY5Y cells were seeded and differentiated as described above and treated with TBHP at concentrations of 10, 20, 30, 50, and 100 μM for 24 h.

Following treatment, a fresh 12 mM MTT stock solution was prepared in PBS. The culture medium was discarded, and fresh medium supplemented with 10% MTT solution (110 μL) was added to each well. Cells were incubated at 37 °C for 2 h. After incubation, the medium was discarded, and 50 μL of DMSO was added to dissolve the formazan crystals. Plates were placed on a shaker and incubated at 37 °C for 10 min. Absorbance was measured at 570 nm using a microplate reader (BioTek Synergy HT, Winooski, VA, USA), with blank wells serving as controls. Cell viability was normalized to DMSO-treated control cells, which were set to 100%.

### 2.3 Pharmacological inhibition of FTO

MO-I-500 was synthesized as previously described (Zheng et al., 2014), dissolved in DMSO to a stock concentration of 10 mM, and further diluted in DMSO to a working concentration of 1 μM. After differentiation, the cells were washed and incubated at 37 °C for 72 h in serum-free DMEM/F-12 medium supplemented with the inhibitor.

For the MO-I-500 treatment group, dSH-SY5Y cells were exposed to the FTO inhibitor alone for 72 h. For the MO-I-500 + TBHP (25 or 50 μM) groups, dSH-SY5Y cells were first pretreated with 1 μM MO-I-500 for 48 h and then co-treated with MO-I-500 and 25 or 50 μM TBHP for an additional 24 h, for a total treatment duration of 72 h. The control group cells were treated with 0.1% DMSO.

### 2.4 FTO siRNA down-regulation

FTO knockdown was achieved using the Lipofectamine™ RNAiMAX Transfection Protocol. Differentiated SH-SY5Y (dSH-SY5Y) cells were transfected with anti-FTO siRNAs (Ambion™ FTO Silencer™ Select Pre-Designed siRNA, Cat# 439220, ID# 33510 and ID# 35512) or Silencer™ Select Control siRNA. Transfections were performed using Lipofectamine™ RNAiMAX Transfection Reagent (Cat# 13778150, Invitrogen) according to the manufacturer’s specifications.

After differentiation, the spent media was replaced with fresh serum-free DMEM/F-12 medium. A siRNA-Lipofectamine complex was prepared in Opti-MEM (Gibco™ Opti-MEM™ I Reduced Serum Medium, Cat# 31985070). Briefly, siRNAs and Lipofectamine were diluted separately in Opti-MEM medium, then combined at a 1:1 ratio to form the siRNA-Lipofectamine complex, which was added to the cells. Cells were incubated with the transfection complex for 48 h at 37 °C. After 48 h, the transfection medium was removed, and the cells were washed and incubated for an additional 24 h in serum-free DMEM/F-12 medium. Silencing efficiency is reported in the supplementary data.

### 2.5 Mitochondrial respiration assessment

Mitochondrial function was measured using the XF24 Seahorse Extracellular Flux Analyzer (Agilent Technologies). SH-SY5Y cells were plated at a density of 4 × 10^4^ cells per well and differentiated (see Cell Cultures and Differentiation) in Agilent Seahorse XF24 cell culture microplates (Seahorse XFe24 FluxPak, Cat# 102340–100). The effects of FTO on energy metabolism were assessed using pharmacological inhibition or siRNA knockdown in dSH-SY5Y cells. For each experiment, 10 out of 24 wells were assigned to the control group (DMSO-treated cells or siCtrl-transfected cells), and 10 wells to the experimental group (MO-I-500-treated cells or siFTO-transfected cells).

Mitochondrial respiration was measured using the Agilent Seahorse XF Cell Mito Stress Test. On the day of the assay, the cell growth medium was replaced with Assay DMEM (pH 7.4, adjusted with NaOH) supplemented with 15 mM glucose, 1 mM sodium pyruvate, and 2 mM glutamine. The cell culture microplate was incubated at 37 °C in a non-CO2 incubator for 45 min to 1 h before the assay. The protocol involved three sequential injections of mitochondrial inhibitors: oligomycin (2 μM), FCCP (1.5 μM; optimized for maximum respiratory capacity), and rotenone/antimycin A (2 μM). After basal measurements, each inhibitor was injected, and two to three measurement cycles were performed per injection.

All data were reported as oxygen consumption rate (OCR) values normalized to cell counts.

### 2.6 Mitochondrial superoxide assay

The Invitrogen™ MitoSOX™ Red (MSR) Mitochondrial Superoxide Indicator was used to detect superoxide in the mitochondria of dSH-SY5Y cells. MSR is a live-cell permeant reagent that rapidly and selectively targets mitochondria. Upon oxidation by superoxide, it exhibits bright red fluorescence. The dye stock solution was prepared following the manufacturer’s protocol, and a final concentration of 5 μg/mL was used for cell staining.

Cells were seeded in 12-well plates at a density of 10 × 10^4^ cells per well and differentiated as described above. Fully differentiated cells were treated with MO-I-500 (1 μM) in the MO-I-500 and MO-I-500 + TBHP groups for 72 h. In the TBHP and MO-I-500 + TBHP groups, TBHP was added 24 h before the end of the treatment. The control group was treated with DMSO.

After treatment, dSH-SY5Y cells were trypsinized and incubated with MitoSOX for 30 min in the dark. Cells were then washed twice with PBS and resuspended in HBSS medium. Fluorescence was measured using a BD LSR flow cytometer (BD Biosciences, NJ, USA), and data were analyzed with FlowJo software. Mean Fluorescence Intensities (MFIs) were compared across groups, and the results were expressed as fold change relative to the control group.

### 2.7 Intracellular ATP content

Intracellular ATP content was measured using the ATP Bioluminescence Assay Kit CLS II (Merck, NJ, USA) according to the manufacturer’s protocol. SH-SY5Y cells were seeded in 24-well plates at a density of 6 × 10^4^ cells per well and differentiated as described above. After differentiation, cells were treated with MO-I-500 for 72 h, and TBHP was added to the TBHP and TBHP + MO-I-500 groups 24 h before measurement.

On the day of the experiment, the spent medium was discarded, and cells were washed twice with PBS. Cells were lysed with TE buffer (100 mM Tris, 4 mM EDTA, pH 7.75) on ice. Lysates were transferred to 1.5 mL tubes and heated at 95 °C for 7 min. After incubation, samples were centrifuged at 14,000 rpm for 3 min to pellet cell debris. Each supernatant was mixed with luciferase reagent in a black 96-well plate and analyzed in luminescence mode using a BioTek Synergy HT microplate reader.

### 2.8 Mitochondrial mass and mitochondrial membrane potential (MMP)

MitoTracker™ Green FM (MTG; M7514, ThermoFisher Scientific, MA, USA) and MitoTracker™ Red CMXRos (MTR; M7512, ThermoFisher Scientific) were used to assess changes in mitochondrial mass and mitochondrial membrane potential (MMP) in dSH-SY5Y cells. MTG and MTR probes passively diffuse across the plasma membrane and accumulate in active mitochondria. The dye stock solution was prepared following the manufacturer’s protocol, and a final concentration of 40 nM was used for cell staining.

Cells were seeded in 24-well plates at a density of 6 × 10^4^ cells per well and differentiated as described above. Fully differentiated cells were treated with MO-I-500 (1 μM) in the MO-I-500 and MO-I-500 + TBHP groups for 72 h. In the TBHP and MO-I-500 + TBHP groups, TBHP was added 24 h before the end of the treatment. The control group was treated with DMSO.

Following treatment, dSH-SY5Y cells were trypsinized and stained with MTG and MTR for 30 min in the dark. Stained cells were washed twice with PBS and resuspended in HBSS medium. Fluorescence intensity was measured using a BD LSR flow cytometer (BD Biosciences, NJ, USA), and data were analyzed with FlowJo software. Mean Fluorescence Intensities (MFIs) were compared across groups, and the results were expressed as fold change relative to the control group.

### 2.9 Intracellular ROS measurement

Intracellular ROS levels were measured using 2′,7′-dihydrofluorescein diacetate (DCFDA; Sigma-Aldrich), a ROS-sensitive fluorescent probe. SH-SY5Y cells were cultured and seeded in 24-well plates at a density of 6 × 10^4^ cells per well and differentiated as described above. Fully differentiated cells were treated with MO-I-500 (1 μM) in the MO-I-500 and MO-I-500 + TBHP groups for 72 h. TBHP was added to the TBHP and MO-I-500 + TBHP groups 24 h before the end of the treatment. The control group was treated with DMSO.

Following treatment, the supernatant was discarded, and cells were stained with 10 μM DCFDA for 30 min. After three washes with PBS, cells were observed under a fluorescence microscope (AIF, Arsenal, Czech Republic) at excitation/emission wavelengths of 495 nm/527 nm. Fluorescence intensity was quantified using ImageJ software (v1.53e; Rasband W.S., ImageJ, U.S. NIH, Bethesda, Maryland, USA). For each well, images of DCFDA and DAPI dyes were captured using the appropriate filters. The mean gray values for both dyes were measured using ImageJ software, and the DCFDA/DAPI ratio was subsequently calculated. The results are expressed as a fold change to the control group.

### 2.10 Protein isolation and Western blot analysis

Protein levels were determined by Western blot analysis. Cells were washed with ice-cold PBS, scraped, and harvested by centrifugation at 1,000 × g for 10 min at 4 °C. The resulting pellet was resuspended in TMES buffer (20 mM Tris, 3 mM MgCl2, 1 mM EDTA, 250 mM sucrose, pH 7.4) supplemented with protease inhibitors (cOmplete™ Protease Inhibitor Cocktail, Roche). Cells were homogenized by 20 strokes through a 25G gauge needle and sonicated twice for 10 s using an ultrasonic converter (Bandelin UW 2070). Protein concentration was determined using the Bicinchoninic Acid (BCA) Protein Assay.

Samples were solubilized in Laemmli buffer, and equal amounts of protein were loaded onto 10% sodium dodecyl sulfate–polyacrylamide gels (SDS-PAGE). After electrophoresis, to verify the equal protein loading and transfer, reversible Ponceau S staining was performed.

Proteins were transferred onto nitrocellulose membranes. Membranes were blocked for 1 h at room temperature with 5% (w/v) non-fat dry milk in Tris-buffered saline (10 mM Tris, 150 mM NaCl, pH 8) containing 0.1% (v/v) Tween 20 (TBS-T buffer).

After blocking, membranes were incubated with primary antibodies (Table 1) overnight at 4 °C. Membranes were then washed three times for 10 min each with wash buffer (TBS with 3% Tween 20), followed by incubation with a horseradish peroxidase-labeled secondary antibody for 1 h at room temperature. After three additional washes, protein bands were visualized using enhanced chemiluminescence according to the manufacturer’s instructions. Immunoblots were scanned and quantitatively analyzed using ImageJ software.

**Table 1:** Western blot antibody.

Antigen	Host	Clonality	Dilution	Manufacturer	Catalogue nr
FTO	Mouse	Monoclonal	1:10000	Abcam	ab92821
Anti-PGC1 alpha + beta antibody	Rabbit	Polyclonal	1:1000	Abcam	ab54481
Mouse IgG HRP Linked Whole Ab	Sheep	Unknown	1:10000	GE Healthcare UK limited	NXA931V
Rabbit IgG HRP Linked Whole Ab	Donkey	Unknown	1:20000	Cytiva	NA934-1ML
γ Enolase NSE-P1	Mouse	Monoclonal	1:15000	Santa Cruz	sc-21738
MAP2	Mouse	Monoclonal	1:500	Santa Cruz	sc-74421
SYP (SY38)	Mouse	Monoclonal	1:3000	Santa Cruz	sc-58304

The signal from the target proteins was normalized to the total protein content, as determined by Ponceau S staining.

### 2.11 Statistical analysis

All data are expressed as the mean ± S.E.M. Statistical significance was determined using two-way ANOVA, followed by Sidak’s or Tukey’s multiple comparisons test, performed in Prism software (GraphPad Prism 8.0.1, San Diego, CA, USA). For the MTT assay, statistical significance was assessed using a one-way ANOVA followed by Dunnett’s multiple comparisons test. In Supplementary Figure S2, statistical significance was determined using an unpaired t-test.

The symbols * or # indicate a statistically significant difference in *post-hoc* analysis as follows: */# ~ *p* < 0.05, **/## ~ *p* < 0.01, ***/### ~ *p* < 0.001, ****/#### ~ *p* < 0.0001.

## 3 Results

### 3.1 Effects of MO-I-500 and tert-butyl hydroperoxide (TBHP) on SH-SY5Y cell viability

**Figure 1:**
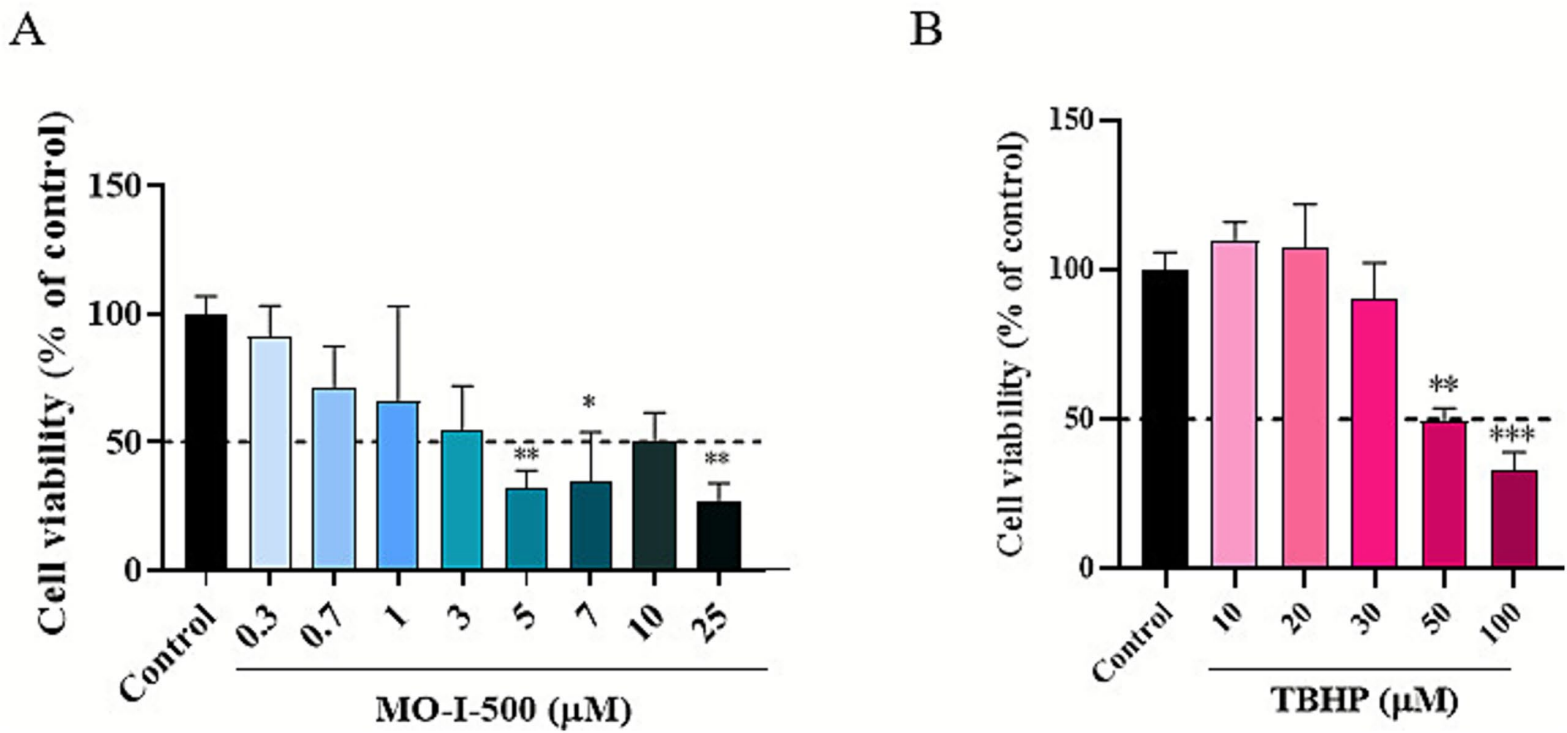
Effects of MO-I-500 and tert-butyl hydroperoxide (TBHP) on dSH-SY5Y cell viability. Cell viability was measured using the MTT assay. SH-SY5Y cells were seeded and differentiated in 96-well plates. Once fully differentiated, cells were treated with MO-I-500 for 72 h. We observed a significant main effect of MO-I-500 (*F* (8, 21) = 4.115, *p* = 0.0044). Dunnet’s post-hoc analysis revealed significant differences at doses 5, 7, 10, and 25 μM. MO-I-500 [1 μM] was identified as a safe dose for pretreatment of dSH-SY5Y cells **(A)**. Subsequently, we treated cells with TBHP for 24 h **(B)**. We observed a significant main effect of TBHP (*F* (5, 12) = 12.77, *p* = 0.0002). Dunnet’s *post-hoc* analysis revealed significant differences at TBHP concentrations of 50 and 100 μM. Thus, TBHP [25 μM] and TBHP [50 μM] were selected to induce dose-dependent oxidative stress. Data are normalized to the control group and expressed as mean ± SEM.

To evaluate the effects of MO-I-500 and TBHP on cell viability, an MTT assay was performed (Figure 1). SH-SY5Y cells were first differentiated and then treated with various concentrations of each compound. For MO-I-500, concentrations of 0.3, 0.7, 1, 3, 5, 7, 10, and 25 μM were tested for 72 h, with DMSO-treated cells serving as controls. MO-I-500 treatment caused a significant reduction in cell viability at concentrations of 5 μM and above (Figure 1A). Therefore, based on these results, 1 μM was selected as a safe dose for subsequent experiments. To assess the cytotoxicity of TBHP, concentrations of 10, 20, 30, 50, and 100 μM were tested for 24 h, with DMSO-treated cells as controls. TBHP reduced cell viability in a dose-dependent manner, with a calculated IC50 of approximately 45 μM (50% cell viability of controls; Figure 1B). Therefore, 25 μM and 50 μM were selected as testing concentrations for future experiments.

### 3.2 Pharmacological inhibition of FTO via MO-I-500 and FTO knockdown reduce mitochondrial respiration in dSH-SY5Y neuron-like cells

**Figure 2:**
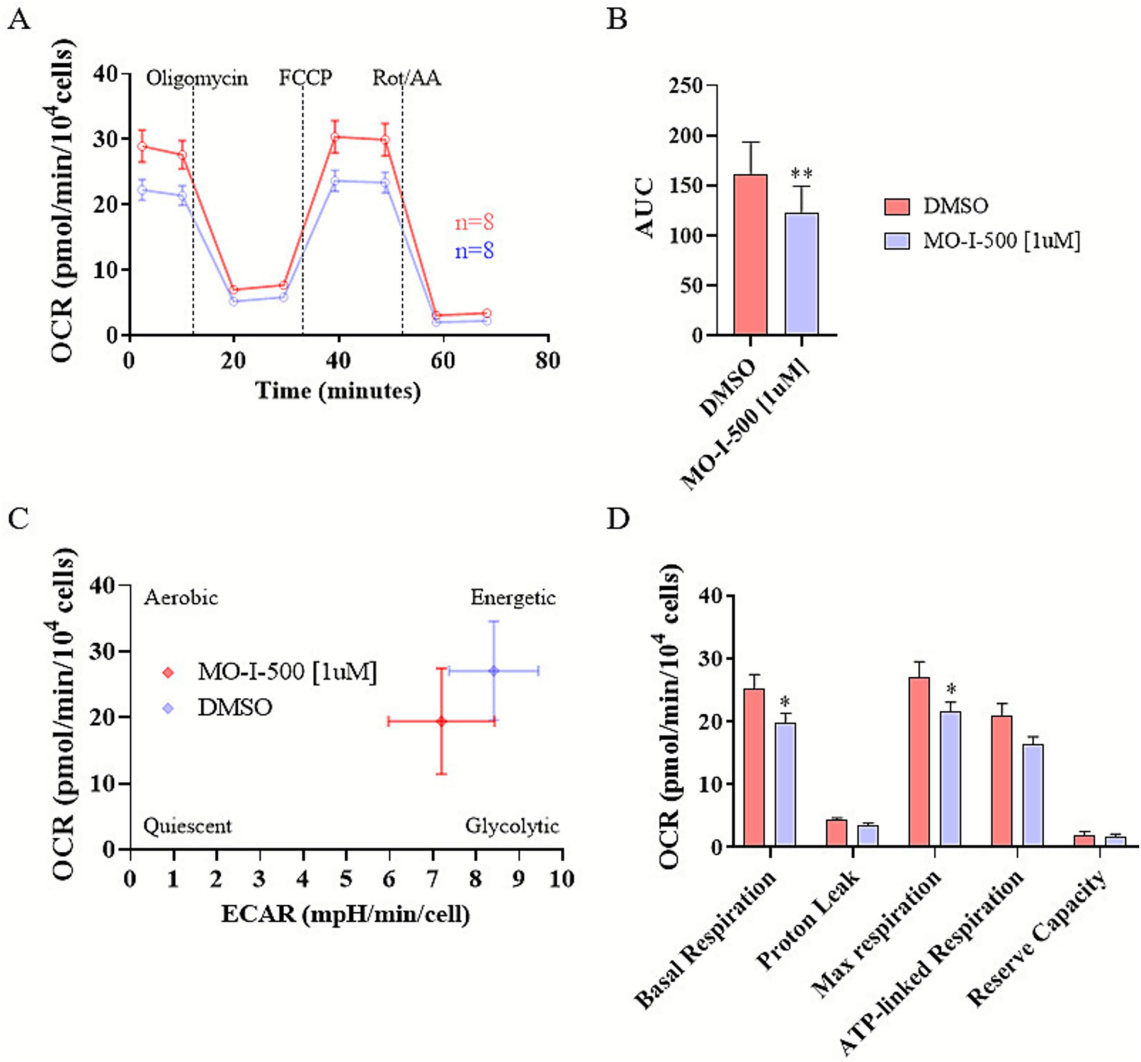
Pharmacological inhibition of FTO via MO-I-500 reduces mitochondrial respiration in dSH-SY5Y neuron-like cells. SH-SY5Y neuroblastoma cells were differentiated and treated with MO-I-500 (1 μM) for 72 h to inhibit FTO demethylase activity. Mitochondrial respiration was assessed using the Seahorse XF Cell Mito Stress Test. Oxygen Consumption Rate (OCR) traces for control cells (DMSO) and MO-I-500-treated cells are shown **(A)**. MO-I-500 treatment significantly reduced mitochondrial respiration compared to control cells **(A,B)**. MO-I-500 treatment changed the metabolic phenotype of dSH-SY5Y cells, enabling them to maintain ATP production **(C)**. Basal and maximal respiration were also significantly decreased in MO-I-500-treated cells as shown by a two-way ANOVA demonstrating significant main effects of MO-I-500 (*F* (1, 70) = 13.37, *p* = 0.0005) and respiratory parameter (*F* (4, 70) = 110.0, *p* < 0,0001), followed by a Sidak’s multiple comparisons *post-hoc* test **(D)**. Data are expressed as mean ± SEM (*n* = 8). Statistical significance was determined using two-way ANOVA followed by Sidak’s multiple comparisons test.

**Figure 3:**
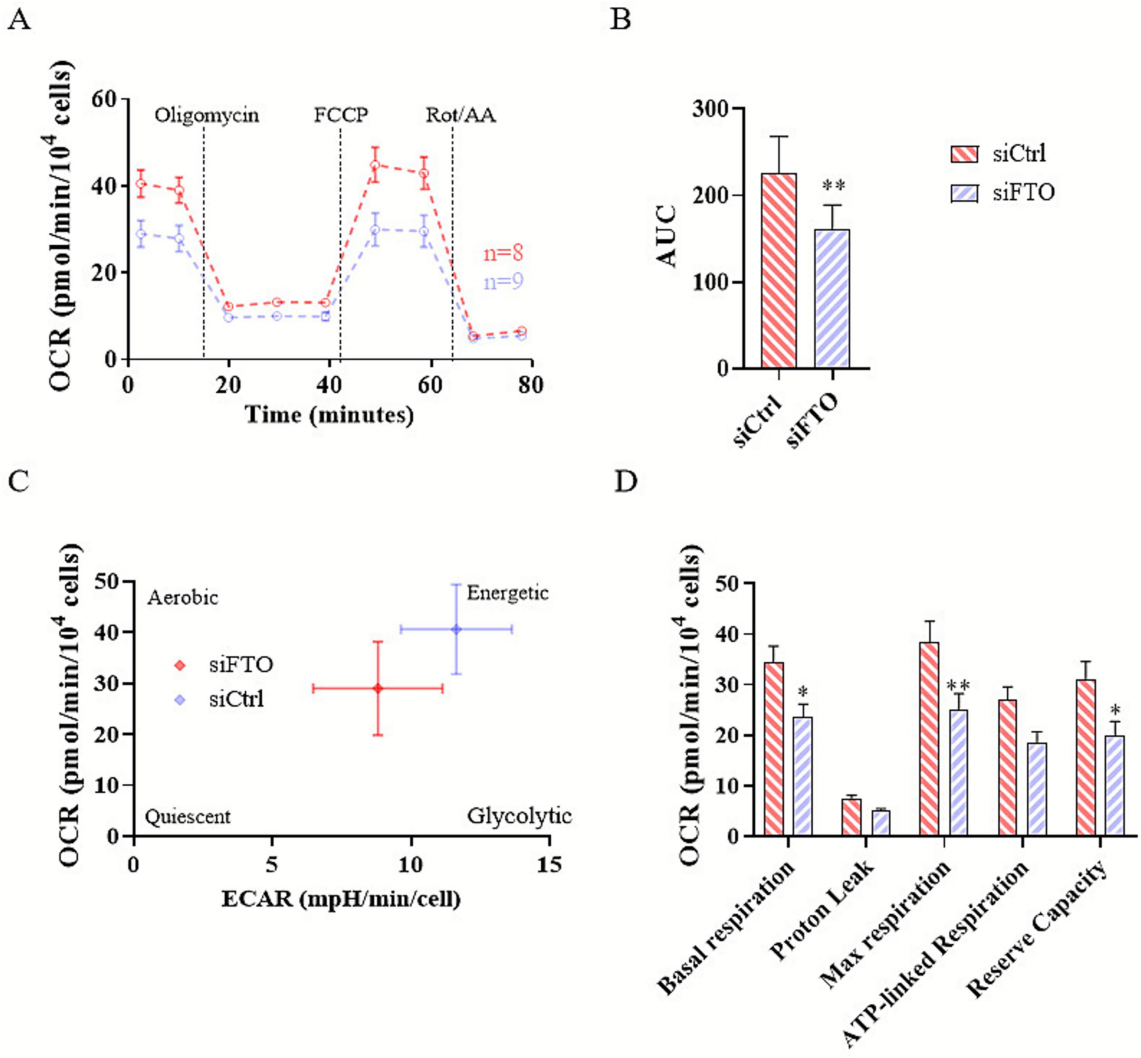
FTO siRNA knockdown reduces mitochondrial respiration in dSH-SY5Y neuron-like cells. SH-SY5Y neuroblastoma cells were differentiated and transfected with siRNAs targeting FTO (siFTO) for 48 h to knock down FTO expression. Mitochondrial respiration was assessed using the Seahorse XF Cell Mito Stress Test. Mitochondrial respiration was significantly reduced in siFTO-transfected cells compared to siCtrl control cells **(A,B)**. FTO siRNA knockdown altered the cellular energetic status, as shown in **(C)**. Specifically, FTO knockdown significantly decreased basal respiration, maximal respiration, and reserve capacity as revealed by a two-way ANOVA showing significant main effects of KNOCKDOWN (*F* (1, 75) = 29.14, *p* < 0.0001) and RESPIRATORY PARAMETER (*F* (4, 75) = 27.10, *p* < 0.0001) and subsequent Sidak’s multiple comparisons *post-hoc* test. **(D)**. Data are expressed as mean ± SEM (*n* = 8, 9). Statistical significance was determined using two-way ANOVA followed by Sidak’s multiple comparisons test.

Alterations in mitochondrial respiration in differentiated SH-SY5Y neuron-like cells were assessed using the Seahorse XF Cell Mito Stress Test after FTO inhibition (Figure 2) or knockdown (Figure 3). SH-SY5Y cells were seeded in Seahorse XF24 V7 PS Cell Culture Microplates at a density of 4 × 10^4^ cells per well and differentiated as described in the Materials and Methods section. Following differentiation, cells were treated with MO-I-500 for 72 h or transfected with FTO-targeting siRNAs for 48 h. Mitochondrial respiration was then measured using the Seahorse XFe24 Analyzer.

Oxygen consumption rate (OCR) revealed that FTO inhibition significantly reduced mitochondrial respiration compared to controls (Figures 2A,B). Control cells exhibited a prominent energetic phenotype, utilizing both mitochondrial respiration and glycolysis, whereas MO-I-500-treated cells shifted to a more energy-efficient metabolic state (Figure 2C). Both basal and maximal respiration were significantly decreased in treated cells, while ATP-linked respiration remained unchanged, suggesting the absence of mitochondrial damage (Figure 2D). These findings suggest that the reduction in mitochondrial respiration reflects metabolic optimization rather than mitochondrial dysfunction.

Similarly, FTO knockdown significantly decreased mitochondrial respiration compared to controls (Figures 3A,B) and induced a shift to an energy-efficient phenotype (Figure 3C). Basal respiration, maximal respiration, and reserve capacity were all significantly reduced, but no significant differences were observed in ATP-linked respiration (Figure 3D). These results support the notion that the observed decrease in mitochondrial respiration represents a metabolic adaptation that maintains ATP levels while reducing energy demand, potentially promoting cellular resilience and neuroprotection under oxidative stress conditions.

### 3.3 MO-I-500 significantly reduced TBHP-induced oxidative stress in dSH-SY5Y neuron-like cells

**Figure 4:**
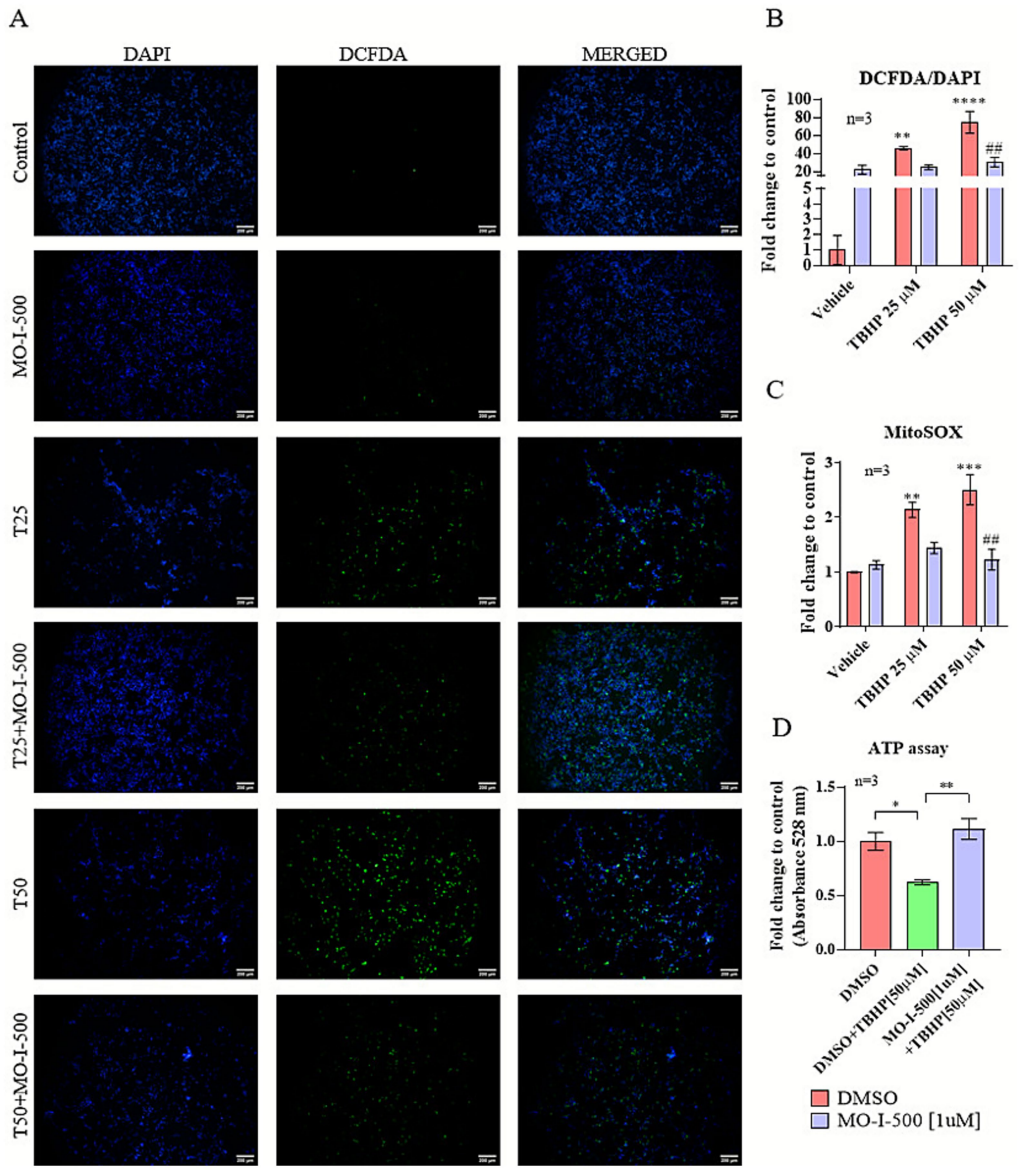
FTO inhibition via MO-I-500 significantly reduces TBHP-induced oxidative stress in dSH-SY5Y cells. Intracellular ROS levels were determined by DCFDA/DAPI staining. dSH-SY5Y neuroblastoma cells were treated with MO-I-500 (1 μM) only for 72 h to inhibit FTO demethylase activity or pretreated with MO-I-500 for 48 h followed by co-treatment with MO-I-500 and TBHP (25 μM or 50 μM) for an additional 24 h. After treatment, cells were stained with DCFDA (10 μM) for 30 min and observed under a fluorescence microscope. Representative images of DCFDA staining (scale bar = 200 μm). Fluorescence intensity, reflecting intracellular ROS levels, was quantified using ImageJ software. Mean gray values of DCFDA and DAPI were measured in ImageJ software and the ratio DCFDA/DAPI was calculated and expressed as fold change to control cells **(A)**. A two-way ANOVA revealed significant main effects of MO-I-500 (*F* (1, 12) = 9.174, *p* = 0.0105), TBHP (*F* (2, 12) = 23.82, *p* < 0.0001), as well as significant interaction (F (2, 12) = 15.41, *p* = 0.0005). Thus, TBHP treatment significantly increased intracellular ROS levels in a dose-dependent manner (25 μM and 50 μM TBHP vs. DMSO control). Pre-treatment with MO-I-500 significantly reduced ROS levels **(B)**. Superoxide levels were further assessed by flow cytometry using the MitoSOX™ Red indicator. MFIs were compared across groups, and the results were expressed as fold change relative to the control group. A two-way ANOVA revealed significant main effects of MO-I-500 (*F* (1, 11) = 23.03 *p* = 0.0006), TBHP (*F* (2, 11) = 16.44, *p* = 0.0005), as well as significant interaction (F (2, 11) = 10.02, *p* = 0.0033). TBHP treatment thus significantly increased superoxide levels compared to the DMSO control group, while pre-treatment with MO-I-500 significantly reduced superoxide levels compared to both TBHP [25 μM] and TBHP [50 μM]. This reduction was statistically significant when comparing MO-I-500 + TBHP [50 μM] to TBHP [50 μM] **(C)**. ATP levels were measured using the ATP Bioluminescence Assay Kit. Under normal conditions, ATP content was not significantly different between DMSO and MO-I-500-treated cells. However, under oxidative stress conditions, ATP levels were significantly reduced after TBHP treatment but restored by pre-treatment with MO-I-500. A one-way ANOVA revealed a significant main effect of MO-I-500 (*F* (2, 6) = 12.19, *p* = 0.0077), Tukey’s *post-hoc* test showed significant differences between the DMSO+TBHP group and the remaining groups **(D)**. Data are expressed as mean ± SEM (*n* = 3). The symbol # denotes differences as compared to the negative control (Vehicle/DMSO group). The symbol * denotes differences between the MO-I-500-treated and DMSO-treated group within the same TBHP concentration.

To evaluate the cytoprotective effects of FTO inhibition under stress conditions, oxidative stress was induced in dSH-SY5Y cells using TBHP. Cells were pre-treated with MO-I-500 followed by co-treatment with TBHP (Figure 4).

Representative images of DCFDA/DAPI staining for each treatment condition are shown in Figure 4A to illustrate the qualitative differences in ROS levels before quantitative analysis.

To first assess oxidative damage, intracellular ROS levels were measured by fluorescence microscopy (Figure 4B). dSH-SY5Y cells were stained with DCFDA and DAPI following MO-I-500 and TBHP treatment, and mean gray values were quantified using ImageJ. The DCFDA/DAPI ratio was calculated and expressed as fold change relative to the control group. TBHP promoted dose-dependent ROS production compared to the vehicle group (TBHP [25 μM] vs. Vehicle, *p* < 0.01; TBHP [50 μM] vs. Vehicle, *p* < 0.0001). MO-I-500 pre-treatment significantly reduced ROS levels compared to the TBHP [50 μM] group (p < 0.01). Although Vehicle-DMSO and Vehicle-MO-I-500 appear visually different, statistical analysis did not reveal a significant difference between these two groups. The two-way ANOVA with Tukey’s post-hoc test confirmed that the Vehicle groups did not differ significantly (*p* = 0.1989). The lack of significance is primarily due to high variability within the Vehicle + DMSO replicates and the small sample size (*n* = 3), which reduced statistical power.

Superoxide levels were then analyzed using the MitoSOX probe and flow cytometry (Figure 4C). Mean fluorescence intensities were compared across groups and expressed as fold change relative to the control. TBHP caused a significant increase in superoxide levels (TBHP [25 μM] vs. Vehicle, *p* < 0.001; TBHP [50 μM] vs. Vehicle, p < 0.0001), whereas MO-I-500 pre-treatment counteracted this effect (TBHP [50 μM] vs. MO-I-500 + TBHP [50 μM], *p* < 0.01).

Finally, to evaluate the functional consequences of oxidative stress and its attenuation by FTO inhibition, ATP content was measured using the ATP Bioluminescence Assay Kit (Roche) (Figure 4D). SH-SY5Y cells were seeded at a density of 6 × 10⁴ cells per well and differentiated as described above. After differentiation, cells were treated with MO-I-500 for 72 h, and TBHP (50 μM) was added to the TBHP and TBHP + MO-I-500 groups 24 h prior to measurement. Under basal conditions, ATP content did not differ between vehicle-treated and MO-I-500-treated cells. However, TBHP significantly reduced ATP levels (TBHP vs. Vehicle, *p* < 0.05). MO-I-500 pre-treatment mitigated this decline, significantly increasing ATP content compared to the TBHP group (*p* < 0.01).

### 3.4 Effects of MO-I-500 on mitochondrial mass and mitochondrial membrane potential in dSH-SY5Y cells under stress conditions

**Figure 5:**
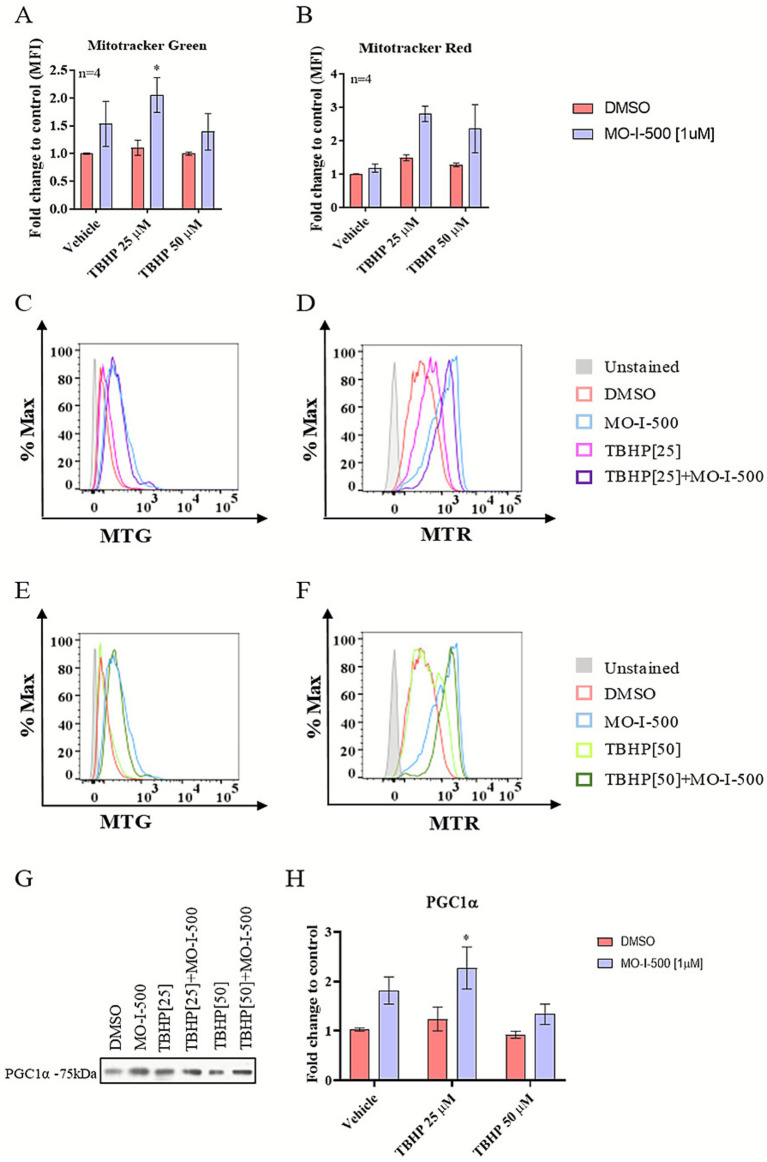
Effects of MO-I-500 on mitochondrial mass and mitochondrial membrane potential in dSH-SY5Y cells under stress conditions. Mitochondrial mass and mitochondrial membrane potential were evaluated via flow cytometry using MitoTracker™ Green FM (MTG) and MitoTracker™ Red CMXRos (MTR), respectively. dSH-SY5Y neuroblastoma cells were treated with MO-I-500 (1 μM) only for 72 h to inhibit FTO demethylase activity or pretreated with MO-I-500 for 48 h followed by co-treatment with MO-I-500 and TBHP (25 μM or 50 μM) for an additional 24 h. After treatment, cells were trypsinized and stained with MTG and MTR at a final concentration of 40 nM for 30 min at 37 °C. Stained cells were washed and analyzed using a BD LSRII Flow Cytometer. MFIs were compared across groups, and the results were expressed as fold change relative to the control group. FTO inhibition significantly increased mitochondrial mass following MO-I-500 [1 μM] treatment. A two-way ANOVA revealed a significant main effect of MO-I-500 (*F* (1,18) = 9.064, *p* = 0.0075), and Sidak’s multiple comparisons post-hoc test confirmed a significant increase at 25 μM TBHP **(A)**. Although a two-way ANOVA also indicated a significant main effect of MO-I-500 on mitochondrial membrane potential (*F*(1,18) = 7.624, *p* = 0.0129), Sidak’s *post hoc* test did not detect a statistically significant difference between treatment groups **(B)**. Representative histograms of MTG and MTR staining are shown in **(C–F)**. Flow cytometry data were analyzed using FlowJo software. As determined by Western blot analysis, the changes in mitochondrial mass were associated with significant upregulation of PGC-1α expression, demonstrating increased mitochondrial biogenesis. For Western blot analysis, PGC-1α expression was normalized to total protein concentration and expressed as a percentage of the DMSO control. A two-way ANOVA revealed significant main effects of MO-I-500 (F (1, 12) = 13.82, *p* = 0.0029) on PGC-1α protein expression. Sidak’s multiple comparisons *post-hoc* test revealed a significant difference at 25 μM TBHP **(G,H)**. Data are expressed as mean ± SEM (*n* = 3, 4). Levels of statistical significance are denoted as follows: * ~ *p* < 0.05, ** ~ *p* < 0.01, *** ~ *p* < 0.001, **** ~ *p* < 0.0001.

To investigate the effects of MO-I-500 on mitochondrial function under TBHP-induced oxidative stress, mitochondrial mass and mitochondrial membrane potential were evaluated via flow cytometry using MitoTracker Green and Red probes (Figure 5). MFIs were compared across groups, and the results were expressed as fold change relative to the control group.

Pretreatment with MO-I-500 showed a significant increase in mitochondrial mass, whereas no statistically significant change was observed in mitochondrial membrane potential (Figures 5A,B). Representative histograms depicting the fluorescence distributions for MitoTracker Green and MitoTracker Red are shown in Figures 5C–F, illustrating the shifts in mitochondrial mass and membrane potential across treatment groups. Although MitoTracker Green and MitoTracker Red are commonly used in flow cytometry to assess mitochondrial content and membrane potential, respectively, both dyes have known limitations under oxidative stress conditions. In this study, MTG and MTR signals were therefore interpreted as relative indicators rather than absolute measures of mitochondrial mass. In the absence of an increase in MTR, we interpreted the elevation in MTG fluorescence as a relative increase in mitochondrial content, an interpretation further supported by increased expression of PGC-1α.

To confirm the role of FTO inhibition in mitochondrial biogenesis, the expression of the transcriptional co-activator peroxisome proliferator-activated receptor gamma coactivator 1-alpha (PGC-1α) was assessed by Western blotting. Pretreatment with MO-I-500 significantly upregulated PGC-1α expression after TBHP [25 μM] treatment (TBHP [25 μM] vs. MO-I-500 + TBHP [25 μM], *p* < 0.05; Figures 5G,H), supporting the role of FTO in regulating mitochondrial biogenesis under oxidative stress conditions.

## 4 Discussion

In this study, we investigated the effects of the small-molecule FTO inhibitor MO-I-500 under both normal and oxidative stress conditions in retinoic acid (RA) and BDNF-differentiated SH-SY5Y neuronal cells (dSH-SY5Y).

Under normal conditions, FTO inhibition or knockdown led to a significant reduction in mitochondrial respiration, as indicated by decreased oxygen consumption rate (OCR), suggesting a metabolic shift toward a more energy-efficient state. Notably, ATP-linked respiration and intracellular ATP levels remained largely unchanged, indicating preserved mitochondrial function and energy production despite reduced overall respiration. Control neurons exhibited a prominent energetic phenotype, utilizing both mitochondrial respiration and glycolysis, whereas MO-I-500-treated or FTO knockdown neurons displayed decreased basal and maximal respiration, consistent with reduced energy demand rather than mitochondrial dysfunction. These findings align with recent evidence that neurons possess intrinsic metabolic flexibility. Neurons are capable of metabolic rewiring, engaging alternative pathways such as anaplerosis to maintain ATP production despite reduced respiratory flux (Motori et al., 2020). Similarly, neurons can adapt to altered energy substrate availability by sustaining oxidative metabolism and network activity under moderate stress (Chausse et al., 2024). Together, these studies support the notion that reduced mitochondrial respiration, as observed following FTO inhibition or knockdown, does not necessarily indicate impaired mitochondrial function but rather reflects a strategic energy optimization. By lowering overall respiratory flux while preserving ATP production, neurons may reduce reactive oxygen species (ROS) generation and maintain bioenergetic homeostasis, enhancing resilience to metabolic and oxidative stress.

Under oxidative stress induced by TBHP, treatment with MO-I-500 resulted in decreased reactive oxygen species (ROS) and superoxide levels, increased ATP production, and enhanced mitochondrial biogenesis, as evidenced by the upregulation of peroxisome proliferator-activated receptor gamma coactivator 1-alpha (PGC-1α). Notably, PGC-1α expression increased at 25 μM TBHP but not at 50 μM, whereas ROS reduction and ATP preservation remained robust at the higher stress level. This distinction highlights the relative contributions of PGC-1α-mediated biogenesis under mild stress versus alternative protective mechanisms under more severe stress. Previous studies suggest that PGC-1α activation is preferentially engaged under mild cellular stress, while alternative pathways dominate under more severe conditions. For instance, Aquilano et al. (2013) showed that p53 regulates PGC-1α expression and antioxidant activity specifically under moderate redox and metabolic imbalance in SH-SY5Y cells. Consistent with this, our findings support PGC-1α as a mediator of mild-stress adaptation, while ATP preservation and ROS suppression at higher TBHP concentrations occur through PGC-1α-independent mechanisms.

AMPK, as an energy sensor influenced by the AMP/ATP ratio, plays a central role in this stress-response (Hardie, 2011). Under mild stress, AMPK promotes PGC-1α activation via transcriptional regulation (CREB, MEF2, SIRT1) and direct phosphorylation, enhancing mitochondrial biogenesis (Cantó and Auwerx, 2009; Rius-Pérez et al., 2020; Chen et al., 2025). Under higher stress, AMPK maintains ATP levels by inhibiting energy-consuming processes (e.g., protein synthesis via mTOR), stimulating catabolic pathways, and promoting autophagy (Garcia and Shaw, 2017). Additional protective mechanisms, such as Nrf2-mediated antioxidant defense (D’Egidio et al., 2025), SIRT3-dependent maintenance of mitochondrial efficiency (Trinh et al., 2024), and downregulation of ion channel activity or synaptic transmission, further support energy homeostasis under severe stress. Therefore, severe oxidative or metabolic stress can uncouple AMPK from PGC-1α by inhibiting transcription factors (MEF2C, FoxO3a), reducing SIRT1 activity through NAD^+^ depletion, or promoting GSK-3β-mediated degradation (Chen et al., 2025), explaining the absence of PGC-1α induction at 50 μM TBHP while ROS suppression and ATP preservation are maintained.

Oxidative stress is a major risk factor for aging and age-related diseases, resulting from an imbalance between ROS generation and antioxidant defenses (Kandola et al., 2015). Excessive ROS overwhelms antioxidant capacity, triggering oxidative damage and leading to mitochondrial dysfunction (Houldsworth, 2024). Mitochondrial dysfunction manifests as impaired biogenesis, disrupted fusion and fission mechanisms, altered mitochondrial morphology, reduced membrane potential, and decreased ATP production (Guo et al., 2013). Together, these abnormalities contribute to cell death, exacerbating the aging process (Misrani et al., 2021; Somasundaram et al., 2024). Reducing oxidative stress to minimize mitochondrial damage is, therefore, a promising therapeutic strategy for aging and age-related diseases (Elfawy and Das, 2019; Lee et al., 2023).

FTO demethylase has been implicated in oxidative stress regulation in the brain. For instance, FTO inhibits oxidative stress by regulating Nrf2 expression in models of cerebral ischemia/reperfusion injury (Hou et al., 2023) and modulates neuronal oxidative stress through ATF3 regulation in an m6A-dependent manner (Zhou et al., 2024). Additionally, we previously showed that FTO downregulation reduced ROS production in LPS-treated primary suprachiasmatic nucleus (SCN) cells (Filipovská et al., 2024) and that MO-I-500 counteracted streptozotocin-induced oxidative damage in astrocytic cells (Cockova et al., 2021). Here, we demonstrate that MO-I-500 also exerts antioxidant properties in neuronal cells, protecting dSH-SY5Y cells from TBHP-induced oxidative damage. TBHP treatment caused a dose-dependent increase in intracellular ROS levels, while MO-I-500 pre-treatment significantly reduced ROS levels and oxidative damage. These effects in neuronal cells, combined with MO-I-500’s protective effects in astrocytes, may provide a dual mechanism to mitigate neurodegeneration. Importantly, a recent study showed that enhancing glucose metabolism and lactate transfer in astrocytes improves synaptic plasticity and cognitive function in models of Alzheimer’s disease (Minhas et al., 2024). Targeting FTO with MO-I-500 could thus achieve simultaneous protective effects in both astrocytes and neurons, offering a novel approach to combat age-associated neurodegenerative diseases.

ROS-driven oxidative damage leads to mitochondrial dysfunction, ultimately causing ATP depletion (Błaszczyk, 2020). FTO has been implicated as a regulator of mitochondrial function, though its effects appear to be cell-type dependent. In hepatocytes, FTO expression reduces mitochondrial content and ATP levels, influencing fat metabolism (Kang et al., 2018). In myotubes, FTO downregulation suppresses mitochondrial biogenesis and energy production, reducing mtDNA-encoding gene and PGC-1α expression while lowering ATP levels (Wang et al., 2017). In cardiomyocytes, FTO knockdown disrupts glycolytic capacity and ATP production under Angiotensin II stimulation, aggravating contractile dysfunction (Zhang et al., 2021). In this study, FTO inhibition in dSH-SY5Y cells under normal conditions did not affect ATP production. However, under oxidative stress, MO-I-500 pre-treatment increased ATP production, mitochondrial mass, and membrane potential. Similarly, in STZ-treated astrocytes, MO-I-500 partially restored mitochondrial membrane potential and electron transport chain capacity (Cockova et al., 2021). Conversely, in rat cardiomyocytes under acute hypoxic conditions, MO-I-500 increased lactate dehydrogenase release and reduced cell viability (Benak et al., 2023). These findings highlight the importance of investigating cell-type-specific FTO mechanisms and the systemic effects of FTO inhibition.

Evidence suggests that FTO regulates mitochondrial biogenesis through the mTOR-PGC-1α pathway. For example, during myoblast differentiation, FTO mediates mTOR-dependent PGC-1α expression, a key regulator of mitochondrial biogenesis (Wang et al., 2017). In clear cell renal cell carcinoma, FTO restores mitochondrial activity and reduces oxidative stress, impairing tumor growth via the FTO-PGC-1α signaling axis (Zhuang et al., 2019). In this study, we show that under oxidative stress conditions, FTO inhibition increases mitochondrial mass and enhances PGC-1α expression, supporting the hypothesis that mitochondrial biogenesis is modulated by an FTO-PGC-1α pathway. Further research is needed to elucidate the mechanisms by which FTO controls PGC-1α expression and mitochondrial biogenesis.

In conclusion, MO-I-500, a novel FTO demethylase inhibitor, modulates the energy metabolism of dSH-SY5Y cells, inducing a metabolic shift that enhances resilience to oxidative stress. Specifically, MO-I-500 mitigated TBHP-induced oxidative stress by reducing intracellular ROS levels, increasing mitochondrial function, and upregulating PGC-1α expression. These findings reveal a role for FTO in regulating mitochondrial biogenesis and suggest MO-I-500 as a promising therapeutic candidate for treating aging and age-related diseases. Together with its demonstrated protective effects in astrocytes, MO-I-500’s dual action on neurons and astrocytes positions it as a unique therapeutic strategy for combating neurodegeneration. Further investigations are needed to confirm these results in animal models and assess the systemic effects of FTO inhibition. Nevertheless, MO-I-500’s ability to cross the blood–brain barrier makes it a strong candidate for further pre-clinical development.

Taken together, our findings demonstrate that FTO inhibition counteracts the bioenergetic consequences of neural aging, presenting a potential therapeutic strategy for age-related neurological disorders.

## Data Availability

The raw data supporting the conclusions of this article will be made available by the authors, without undue reservation.

## Supplementary material

Supplementary Figure S1 siRNA knockdown efficiency. The efficiency of siRNA-mediated FTO knockdown was confirmed by Western blot analysis **(A–C)**. Results showed that FTO expression was significantly reduced compared to non-specific siRNA controls (siCtrl) **(A)**. Unpaired t-test revealed a significant difference (*p*= 0.0463) as denoted by * symbols. Data are expressed as mean ± SEM (*n* = 3).

Supplementary Figure S2 Optimization of SH-SY5Y cell differentiation protocol. **(A)** Representative images of non-differentiated cell (NC, in the upper panel) and differentiated cells (DC, lower panel) at 10X (left) and 20X (right) magnification. Representative images of western blotting analysis **(B–D)**. The expression of neural markers MAP2, SY38 and NSE significantly increased in DC cells compared to NC cells **(E–G)**. Unpaired t-test was performed to determine the statistical significance.
