# Supplementary figures and images for: Small molecule FTO inhibitor MO-I-500 protects differentiated SH-SY5Y neuronal cells from oxidative stress
Source: Front Mol Neurosci. 2026 Jan 12;18:1736173. doi: 10.3389/fnmol.2025.1736173 (PMC12832913; doi:10.3389/fnmol.2025.1736173)

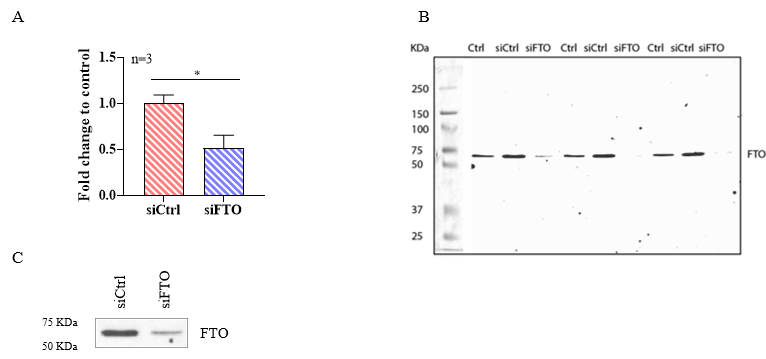

Supplement: Supplementary Figure S1 — siRNA knockdown efficiency. The efficiency of siRNA-mediated FTO knockdown was confirmed by Western blot analysis (A–C). Results showed that FTO expression was significantly reduced compared to non-specific siRNA controls (siCtrl) (A). Unpaired t-test revealed a significant difference (p= 0.0463) as denoted by * symbols. Data are expressed as mean ± SEM (n = 3). [file Image_1.TIF]

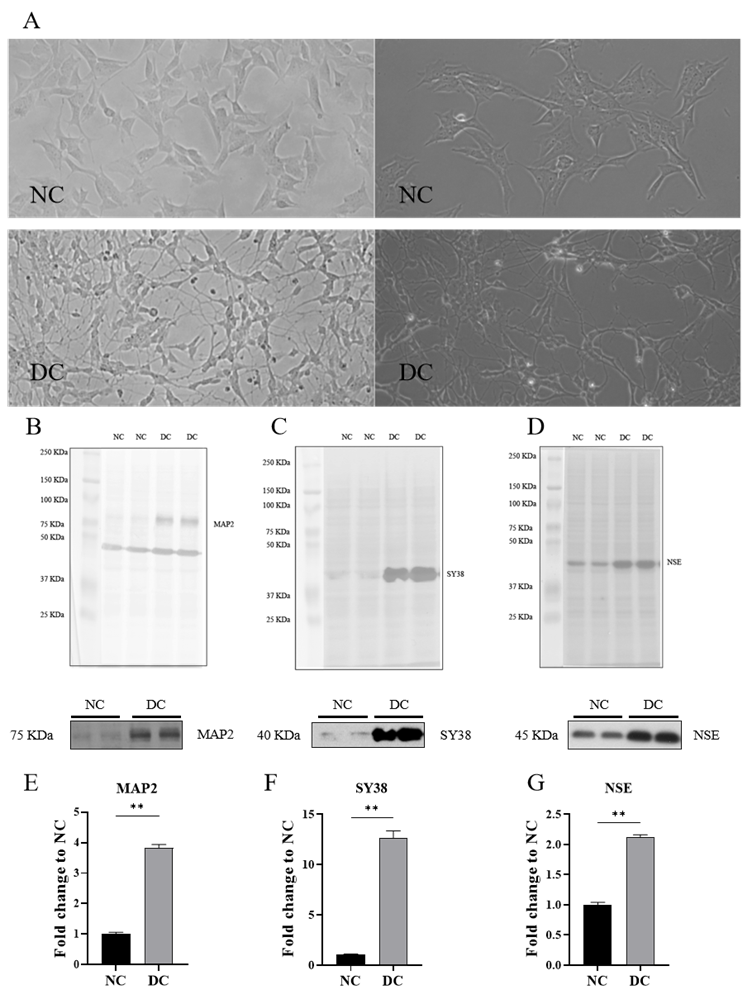

Supplement: Supplementary Figure S2 — Optimization of SH-SY5Y cell differentiation protocol. (A) Representative images of non-differentiated cell (NC, in the upper panel) and differentiated cells (DC, lower panel) at 10X (left) and 20X (right) magnification. Representative images of western blotting analysis (B–D). The expression of neural markers MAP2, SY38 and NSE significantly increased in DC cells compared to NC cells (E–G). Unpaired t-test was performed to determine the statistical significance. [file Image_2.TIF]
